# Awareness of Gestational Diabetes Mellitus Maternal and Neonatal Outcomes Among Women Attending Primary Healthcare Centers in Qassim, Saudi Arabia

**DOI:** 10.7759/cureus.59345

**Published:** 2024-04-30

**Authors:** Nouf S Almazyad, Saulat Jahan

**Affiliations:** 1 Family Medicine Academy, Qassim Health Cluster, Buraidah, SAU

**Keywords:** primary healthcare, saudi arabia, questionnaire, survey, gestational diabetes mellitus, cross-sectional studies

## Abstract

Background

Gestational diabetes mellitus (GDM), diagnosed during pregnancy, can harm both mothers and neonates. GDM awareness among women varies among various countries. Understanding the level of awareness is vital for designing effective health interventions.

Objectives

This study aimed to evaluate GDM awareness among married females at primary healthcare centers (PHCCs) in Qassim, Saudi Arabia, focusing on knowledge regarding adverse maternal and fetal outcomes of GDM.

Methods

An observational cross-sectional study was conducted among married females at PHCCs in Qassim, from June 2023 to October 2023. A two-stage cluster sampling method was used. Four PHCCs were selected in the first stage, and study participants were selected from these centers in the second stage. A self-administered questionnaire was used. Statistical Product and Service Solutions (SPSS, version 23; IBM SPSS Statistics for Windows, Armonk, NY) was used for statistical analysis.

Results

Of the 270 participants, the majority (72.2%) demonstrated ‘poor’ knowledge about GDM adverse outcomes for both mothers and neonates, 17.8% demonstrated a ‘fair’ level, and only 10% displayed a ‘good’ knowledge. Participants’ educational level, personal history of diabetes, and age were associated with knowledge levels. Awareness of specific outcomes related to GDM, both maternal and neonatal, varied among participants. Information on GDM was mainly obtained from mass media and personal networks, while healthcare providers were reported as the least common source.

Conclusion

Based on the results of our study, we conclude that educational interventions, especially involving healthcare providers, are essential to improve awareness about GDM adverse outcomes. Strategies involving educational sessions by healthcare providers and health education materials at PHCCs can improve awareness leading to effective management of GDM and improved maternal and neonatal outcomes.

## Introduction

The American Diabetes Association (ADA) defines gestational diabetes mellitus (GDM) as diabetes diagnosed in the second or third trimester of pregnancy that was not an overt diabetes prior to gestation [1]. There are no typical clinical symptoms of GDM, leading to the crucial importance of screening for GDM [2]. Increased risk of obstetrical complications, such as stillbirth, macrosomia, jaundice, neonatal hypoglycemia, and preeclampsia are associated with GDM [3]. Diet and lifestyle could control glucose levels in GDM, leading to lower birth complications [4]. Age, race, ethnicity, body composition, and diagnostic and screening criteria are factors in determining GDM prevalence [5].

Due to the epidemic nature of obesity among women during reproductive age, worldwide GDM prevalence is increasing [6]. Globally, the prevalence of GDM is reported to be 7-10% of all pregnancies worldwide [7]. The prevalence of GDM in the Middle East and North Africa region ranges from 8.4% to 24.5% [8]. In 2018, a study done in Asia showed that Saudi Arabia is the third highest country with a prevalence of GDM reaching 22.9% [9]. Two recent studies published in 2021 reported a high prevalence of GDM, ranging from 21.6% to 32.6%, among Saudi women [10,11].

Uncontrolled hyperglycemia during pregnancy may lead to negative consequences for both the mother and the fetus. Maternal side effects include stillbirth, premature labor, pre-eclampsia, cesarean section, polyhydramnios, urinary tract infection, pyelonephritis, and puerperal sepsis [12], whereas fetal side effects include birth injury, hypoglycemia, hypocalcemia, delayed lung maturity, respiratory distress syndrome, jaundice, and perinatal mortality [13].

Because of the increasing incidence of GDM, it is important to adopt strategies to control this important health problem, which affects both the mother and child. In this context, creating awareness about GDM by educating women is an important intervention. However, before implementing a health education program, it is important to assess the level of awareness of the target population.

Worldwide, various studies were conducted to assess the level of awareness regarding GDM. In 2018, a study conducted in Zambia among pregnant females determined that the level of education and number of births played a role in the difference in knowledge between participants [14]. A study conducted in Uganda in 2021 concluded that there is a low-level awareness about GDM among pregnant women [15], whereas another study done in Sharjah, UAE, among women of childbearing age, reported a high level of awareness regarding GDM [16]. Some studies conducted in Saudi Arabia assessed the awareness of GDM among women. In 2018, 9,002 adult females participated in a cross‑sectional study done in Saudi Arabia. The study showed poor awareness and knowledge about GDM, mainly in the domain of GDM diagnosis [17]. Another study conducted in Jeddah, Saudi Arabia, in 2022 found that 77.8% of married females had poor knowledge [18]. Similar results were also observed among Saudi females who took part in a cross-sectional study conducted in Almadinah Almunawarah, Saudi Arabia, in 2022, demonstrating poor knowledge among the participants [19].

Despite the high prevalence of GDM in Saudi Arabia, there is a dearth of studies estimating the awareness level among females in Saudi Arabia, particularly in Qassim. Determining the level of awareness about GDM is vital for controlling this growing health issue. In this context, the current study aimed to explore awareness about GDM among females in Qassim. The objectives of the study were to determine the level of knowledge regarding adverse maternal and fetal outcomes of GDM and to explore the factors associated with the level of knowledge about GDM adverse outcomes among married females attending primary healthcare centers (PHCCs) in Buraidah, Qassim, Saudi Arabia.

## Materials and methods

Study design and setting

An observational cross-sectional study was conducted during the year 2023. The study was carried out among married Saudi females attending PHCCs in Buraidah, Qassim province, Saudi Arabia. Unmarried and non-Saudi females were excluded from the study.

Sample size and sampling technique

The sample size was calculated by using the OpenEpi sample size calculator (Centers for Disease Control and Prevention, Atlanta, GA). The criterion for level of knowledge was taken from a previously published similar study, which showed that 77% of the study participants had poor knowledge about GDM [18]. With a 95% confidence level and a 5% accepted margin of error, the calculated sample size was 270 women.

Data were obtained by choosing four major PHCCs in Buraidah through convenience sampling. All female, married Saudi patients attending those PHCCs were invited to participate in the study until the sample size was completed.

Data collection

The questionnaire included 31 questions divided into two sections. The first section of the questionnaire included items about participants’ demographic characteristics, parity, pregnancy, family, and personal history of chronic diseases and GDM. The second section included items to assess participants’ knowledge about the effect of GDM on mothers and neonates. Furthermore, it gathered information about the participants’ sources of information regarding GDM. In order to prevent the respondents from guessing, the questions included the “I don’t know” option, in addition the to ‘Yes’ or ‘No’ response.

Data were collected using an Arabic semi-structured, self-administered questionnaire in the form of an electronic survey created using Google Forms. The questionnaire was adapted from a previous study conducted in Saudi Arabia [18]. The questionnaire was piloted with the target population for clarity and understandability. No modifications were required as the respondents found the questionnaire clear and understandable. 

At the PHCC reception desk, all eligible females visiting the PHCC were invited to complete the self-administered questionnaire through Google Forms. The objectives of the study were explained, and informed consent was taken from the study participants before distributing the questionnaire. Data were collected from June 2023 to October 2023.

Statistical analysis

The data from Google Forms was downloaded to Microsoft Excel. All variables were coded, and a codebook was prepared with a description of the variables and corresponding codes. The data were analyzed using the software, Statistical Product and Service Solutions (SPSS, version 23; IBM SPSS Statistics for Windows, Armonk, NY).

All demographic, medical, and gestational characteristics were independent variables. The dependent variable was knowledge of the effects of GDM on mothers and neonates. The questionnaire had 21 questions for assessing knowledge regarding maternal and neonatal outcomes of GDM. For all questions, the answer 'Yes' was the correct answer. The answers 'I don't know' and 'No' were marked as incorrect. The correct answer was awarded a 1 score, while the incorrect answer was given a zero score. Thus, the total possible score for all knowledge questions was 21. The level of knowledge about GDM was reported as ‘good’ knowledge if the study participant correctly responded to more than or equal to 75% of knowledge assessment questions, ‘fair’ if responded to 50%-74%, and ‘poor’ for <50% [18].

Frequencies and percentages were calculated for categorical data. Means with standard deviations were calculated for numerical data. To determine the association of sociodemographic characteristics with the knowledge scores, either the Mann-Whitney U test (for two independent samples) or the Kruskal-Wallis test (for more than two independent samples) was employed. Non-parametric tests were used due to the lack of normal distribution of the knowledge scores. Normal distribution was checked with the Shapiro-Wilk test. A p-value of less than 0.05 was considered statistically significant.

Ethical considerations

The ethical approval was taken from the Qassim Regional Research Ethics Committee (approval number: 607/44/15543). Informed consent was obtained from all participants. The objectives of the study were described to the participants before requesting to respond. It was explained to all participants that they had the right to withdraw from the study at any time without any obligation towards the research team. Access to data was limited to investigators and authorized people. No interventions nor additional investigations were performed.

## Results

The questionnaire was distributed to 300 potential participants attending PHCCs in Buraidah, Qassim Region. Out of these, 270 patients responded and completed the study questionnaire, leading to a response rate of 90%.

A total of 270 married females participated in the study. The age of the respondents ranged from 18 to 67 years, with a mean of 42.35 (±10.3) years. Table 1 shows the demographic profile of the study participants. The majority of participants (n=213, 78.9%) had a university degree and 158 (58.5%) were employed. Most of the participants (n=114, 42.4%) had five or more children, whereas 11.5% had no children. A total of 15 (5.6%) study participants were pregnant at the time of the survey; 46.7% of these pregnant ladies were in the second trimester.

**Table 1:** Socio-demographic characteristics of the study participants

Demographic characteristics		Number	Percentage
Age (n=268)	≤ 30 years	45	16.8%
	31-40 years	79	29.5%
	41-50 years	89	33.2%
	More than 50 years	55	20.5%
Education level (n=270)	Non-Schooled	1	0.4%
	Primary school	2	0.7%
	Middle school	5	1.9%
	High school	38	14.1%
	Diploma/university	213	78.9%
	Postgraduation	11	4.1%
Occupation (n=270)	Student	4	1.5%
	Housewife	74	27.4%
	Employee	158	58.5%
	Health care Employee	9	3.3%
	Retired	25	9.3%
Number of children (n=268)	No children	31	11.5%
	1 to 2 children	44	16.4%
	3 to 4 children	80	29.7%
	5 or more children	114	42.4%
Currently pregnant (n=270)	No	255	94.4%
	Yes	15	5.6%
Gestational age (n=15)	First trimester	5	33.3%
	Second trimester	7	46.7%
	Third trimester	3	20.0%

A total of 131 (41.85%) of the study participants reported a personal history of medical disease, while 191 (70.74%) respondents reported a family history of the disease. The most common personal chronic diseases were DM and hypertension. Similar results were observed with a family history of chronic diseases as the majority of participants had a family history of DM and hypertension.

Table 2 demonstrates the responses of women to information about the maternal outcome of GDM. The highest accuracy in correctly identifying the association between GDM and an increased risk of emergency cesarean section (CS) was noticed as 64.4% responded to the question correctly. Conversely, the lowest accuracy was observed in responses regarding the correlation between GDM and the heightened likelihood of oligohydramnios, with only 18.5% of respondents providing the correct answer.

**Table 2:** Study participants’ responses to queries concerning maternal outcomes associated with gestational diabetes mellitus (GDM) CS, Cesarean section; GDM, gestational diabetes mellitus

Questions About Maternal Outcome	Participants’ Response	Number	Percentage
Do you think GDM increases the risk of instrumental delivery?	No	18	6.7%
	Yes	103	38.1%
	I do not know	149	55.2%
Do you think GDM increases the risk of elective CS?	No	29	10.7%
	Yes	153	56.7%
	I do not know	88	32.6%
Do you think GDM increases the risk of emergency CS?	No	18	6.7%
	Yes	174	64.4%
	I do not know	78	28.9%
Do you think GDM increases the risk of preterm delivery?	No	27	10.0%
	Yes	162	60.0%
	I do not know	81	30.0%
Do you think GDM increases the risk of polyhydramnios?	No	20	7.4%
	Yes	96	35.6%
	I do not know	154	57.0%
Do you think GDM increases the risk of oligohydramnios?	No	36	13.3%
	Yes	50	18.5%
	I do not know	184	68.1%
Do you think GDM increases the risk of induction of labor?	No	30	11.1%
	Yes	88	32.6%
	I do not know	152	56.3%
Do you think GDM increases the risk of postpartum hemorrhage (PPH)?	No	44	16.3%
	Yes	72	26.7%
	I do not know	154	57.0%
Do you think GDM increases the risk of rupture of membranes?	No	33	12.2%
	Yes	68	25.2%
	I do not know	169	62.6%
Do you think GDM increases the risk of placental abruption?	No	32	11.9%
	Yes	79	29.3%
	I do not know	159	58.9%
Do you think GDM increases the risk of preeclampsia?	No	22	8.1%
	Yes	128	47.4%
	I do not know	120	44.4%

Table 3 illustrates the responses of women to information about the neonatal outcome of GDM. It was observed that 68.9% of participants correctly linked the association between GDM and an increased risk of high birth weight, making it the highest correctly identified association between GDM and neonatal outcome. Meanwhile, only 15.2% of the participants knew that GDM increases the risk of low birth weight, revealing that it was the least known association between GDM and neonatal outcome known to the study participants.

**Table 3:** Study participants’ responses to queries concerning neonatal outcomes associated with gestational diabetes mellitus (GDM) GDM, gestational diabetes mellitus; NICU, neonatal intensive care unit

Questions About Maternal Outcome	Participants’ Response	Number	Percentage
Do you think GDM increases the risk of low birth weight?	No	139	51.5%
	Yes	41	15.2%
	I do not know	90	33.3%
Do you think GDM increases the risk of high birth weight?	No	16	5.9%
	Yes	186	68.9%
	I do not know	68	25.2%
Do you think GDM increases the risk of NICU admissions for more than 24 hours?	No	17	6.3%
	Yes	151	55.9%
	I do not know	102	37.8%
Do you think GDM increases the risk of hypoglycemia at birth?	No	44	16.3%
	Yes	62	23.0%
	I do not know	164	60.7%
Do you think GDM increases the risk of hyperbilirubinemia?	No	30	11.1%
	Yes	67	24.8%
	I do not know	173	64.1%
Do you think GDM increases the risk of shoulder dystocia?	No	32	11.9%
	Yes	62	23.0%
	I do not know	176	65.2%
Do you think GDM increases the risk of breech delivery?	No	58	21.5%
	Yes	45	16.7%
	I do not know	167	61.9%
Do you think GDM increases the risk of stillbirth?	No	34	12.6%
	Yes	103	38.1%
	I do not know	133	49.3%
Do you think GDM increases the risk of neonatal death?	No	46	17.0%
	Yes	76	28.1%
	I do not know	148	54.8%
Do you think GDM increases the risk of congenital neonatal anomalies?	No	56	20.7%
	Yes	60	22.2%
	I do not know	154	57.0%

A total of 21 questions were asked regarding maternal and fetal outcomes in GDM. None of the questions were correctly answered by 35 (13%) women, while only two (0.7%) answered all questions correctly, scoring 100%. Around three-fourths (76.7%) of the women were able to answer less than half of the questions correctly.

**Figure 1:**
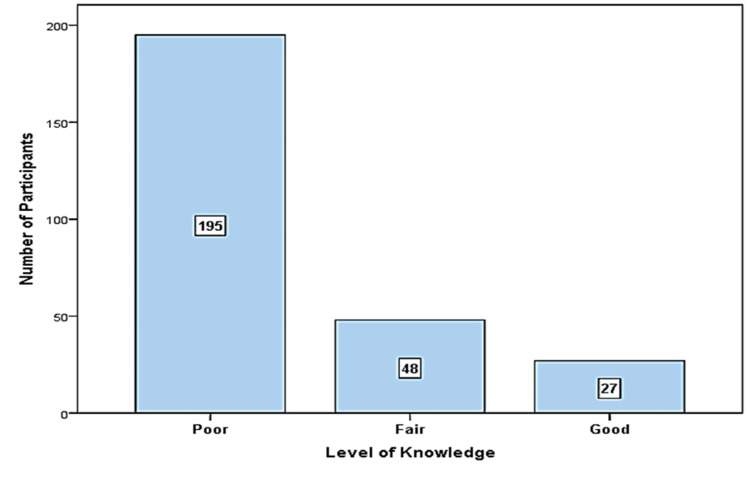
Study participants’ level of knowledge about gestational diabetes mellitus ¶ Level of knowledge: Poor = less than 50% score, Fair = 50%-74% score, Good = 75% and more score

Figure 1 demonstrates the level of knowledge about GDM. Among the respondents, 195 (72.2%) exhibited a poor level of knowledge, 48 (17.8%) demonstrated a fair level, and only 27 (10.0%) displayed a good understanding of GDM.

Out of a total of 21, the overall median knowledge score among study participants was 7 with a minimum of 0 and a maximum of 21. Table 4 shows the association between knowledge scores and socio-demographic characteristics of the study participants. Among the age groups, the knowledge score mean rank was the highest for 31-40 years (155.32), while the lowest for ≤ 30 years (117.47). The difference in knowledge among age groups was statistically significant at p=0.026. The respondents with a personal history of DM had statistically significant (p=0.032) better knowledge as compared to those without a personal history of DM. Although not statistically significant (p=0.083), the mean rank for knowledge scores increased progressively with increasing education: 118.37 for high school, 131.54 for diploma, and notably higher at 176.09 for post-graduation. Among the occupations, the highest mean rank for knowledge scores was observed in healthcare employees. Despite lacking statistical significance, those with a family history of the disease and a family history of DM and GDM had a higher mean rank for knowledge scores as compared to their counterparts. Similarly, those with a personal history of disease, DM, and GDM had higher mean ranks for knowledge scores as compared to those without such histories.

**Table 4:** Association between socio-demographic factors and the knowledge scores among the study participants *Significant at p<0.05, ¶ Kruskal-Wallis test, ∞Mann-Whitney test DM, diabetes mellitus; GDM, gestational diabetes mellitus

Socio-Demographic Factor		N	Mean Rank	P-value
Age in years^¶^ (n=268)	≤ 30	45	117.47	0.026*
	31-40	79	155.32	
	41-50	89	131.67	
	more than 50	55	123.12	
Educational level^¶^ (n=262)	High school	38	118.37	0.083
	Diploma/university	213	131.54	
	Post graduation	11	176.09	
Occupation^¶^ (n=270)	Student	4	98.75	0.091
	Housewife	74	140.30	
	Employee	158	132.35	
	Healthcare employee	9	199.39	
	Retired	25	124.10	
Number of children^¶^ (n=269)	1 to 2 children	44	129.86	0.490
	3 to 4 children	80	144.21	
	5 and more children	114	134.49	
Currently pregnant∞ (n=270)	No	255	136.01	0.565
	Yes	15	126.80	
Family history of disease∞ (n=270)	No	79	123.56	0.105
	Yes	191	140.44	
Family history of DM∞ (n=270)	No	113	133.69	0.747
	Yes	157	136.80	
Family history of GDM∞ (n=270)	No	252	133.06	0.054
	Yes	18	169.61	
Personal history of disease∞ (n=270)	No	157	127.86	0.057
	Yes	113	146.12	
Personal history of DM∞ (n=270)	No	230	131.26	0.032*
	Yes	40	159.88	
Personal history of GDM∞ (n=270)	No	256	134.12	0.212
	Yes	14	160.79	

A Pearson correlation coefficient was computed to assess the association between knowledge regarding maternal outcomes and neonatal outcomes. There was a positive statistically significant correlation between the two variables: r=0.743 and p<0.0001.

The study participants were asked about their source of information regarding GDM. The most common sources of information about GDM were mass media and family/friends, while the least common information source was healthcare providers.

## Discussion

GDM is considered a significant health issue because of its serious complications and high prevalence [3]. Assessing the awareness of married females about GDM and its adverse outcomes on both the mothers and the neonates is essential for educating women and encouraging screening during pregnancy.

In this cross-sectional study, a total of 270 married females were included to assess their knowledge about GDM. Only 10% of the participants demonstrated a good knowledge of GDM, while the majority (72.2%) exhibited poor knowledge, and the remaining (17.8%) had fair knowledge.

Our findings closely align with those of a 2022 study conducted in Jeddah, Saudi Arabia, where 6.1% of Saudi females exhibited excellent awareness of GDM, 77.8% had poor knowledge, and only 16% possessed fair knowledge about GDM [18]. Another study from Al Madinah, also published in 2022, had results similar to our study. The study revealed that 53.45% had a poor level of knowledge, 7.8% exhibited good knowledge, and 38.73% had fair knowledge [19]. In contrast, a study conducted in Sharjah, UAE, among women of childbearing age reported a high level of awareness (73.5%) regarding GDM among women [16]. Similarly, a study carried out in southeast Iran in 2019 concluded that women exhibited good knowledge and positive attitudes toward GDM [20].

In our study, 83% of the respondents had a bachelor's or postgraduate degree. Our findings demonstrated that the higher the educational level the higher the knowledge score. This finding is consistent with the study conducted in Najran, Saudi Arabia, published in 2019, which showed that the level of education played a role in improving knowledge [21]. Additionally, another study highlighted a significant association between educational level and GDM knowledge, revealing an increase in respondents' knowledge of GDM corresponding to higher academic achievements [22].

In the present study, the highest mean rank for knowledge scores was observed among the healthcare employees. This is an expected finding as healthcare workers are supposed to be knowledgeable about various health conditions because of their educational background and work experience.

The current study involved questions about participants' personal and family medical histories. Interestingly, our study noted an increased level of GDM knowledge among females with a personal history of DM. Comparable findings were reported in a 2017 study from the UAE [16]. A better understanding of GDM among individuals with a personal history of DM might be the result of increased exposure to healthcare professionals and a personal inclination to seek information pertaining to diabetes-related conditions.

Variations in knowledge regarding diverse health issues often correlate with an individual's age. Within our study, differences in knowledge levels were noted across various age groups. The mean rank for knowledge scores peaked within the 31-40 years age group, a trend closely resembling findings from a 2016 study in India that reported heightened GDM awareness among females aged 40 years and below [23]. Conversely, the lowest mean rank in our study was observed within the ≤30 years age group. This finding may be attributed to a lack of immediate plans for childbirth among many females in this age group, potentially resulting in a diminished concern or consideration of GDM as an important issue.

Awareness among participants about maternal outcomes related to GDM, particularly risks of various complications, was notably limited. Within our study, awareness among participants regarding maternal outcomes associated with GDM revealed that less than one-third were aware of the potential risks such as increased chances of labor induction, postpartum hemorrhage, rupture of membranes, and placental abruption. A comparable trend was evident in a 2022 study conducted in Jeddah, where also less than one-third of participants exhibited awareness of these aforementioned risks [18]. These findings underscore the imperative need to raise awareness concerning GDM, empowering women to proactively manage their health and promptly seek assistance if signs of any such complications manifest in themselves or others.

Awareness among participants about neonatal outcomes linked to GDM varied significantly, with notable awareness of high birth weight but limited understanding of other associated risks. Regarding neonatal outcomes, our study revealed that approximately 68.9% of participants were aware of the increased risk of high birth weight associated with GDM, marking it as the most accurately answered question concerning neonatal outcomes. Similar outcomes were observed in a 2022 study conducted in Jeddah, where 60.4% of their participants demonstrated awareness of the association between GDM and high birth weight, also marking it as the best-answered question about GDM and neonatal outcomes in their study [18]. Conversely, less than a quarter of our study participants displayed awareness regarding the risks of hyperbilirubinemia, hypoglycemia, and shoulder dystocia among neonates born to mothers with GDM. Emphasizing awareness of these potential complications is crucial, enabling women to effectively manage post-natal issues that may arise.

Information sources on GDM varied among participants, predominantly sourced from mass media and interpersonal circles, while healthcare providers were reported as the least common source. Similar patterns were identified in a 2017 study conducted in Sharjah, where major sources of GDM awareness were family members, media, and educational centers, with healthcare providers contributing to a mere 14% of the informational sources [16]. Consistent outcomes have been reported in other studies as well [24,25]. There is a critical necessity for healthcare providers to assume a more proactive role in augmenting awareness among females regarding GDM. It is imperative to incorporate GDM within health education programs, particularly during the antenatal period, foreseeing improved management and a potential decrease in GDM complications upon enhancing females' education about this condition. To facilitate the dissemination of information, PHCCs could consider displaying posters or infographics pertaining to GDM in waiting areas designated for antenatal care clinics. Additionally, healthcare professionals may organize periodic discussion forums or presentations in universities and other educational institutions to effectively propagate awareness regarding GDM.

Our study had a substantial sample size and focused on a specific target population of married females; however, it has certain limitations. Acknowledging these limitations in the interpretation of study results is crucial for understanding the potential constraints of the findings related to knowledge about GDM among the surveyed population. First, the study might have selection bias as it focuses exclusively on married females attending PHCCs, potentially excluding those who do not visit these centers. Second, the responses might be influenced by social desirability bias or recall bias, affecting the accuracy of self-reported knowledge about GDM. Finally, the findings might not be generalizable to the entire population, as the study sample includes only a specific demographic subset, i.e., married females attending primary healthcare centers in one specific city, and might not reflect the broader population's knowledge about GDM.

## Conclusions

The study revealed a concerning lack of awareness about GDM among married females attending PHCCs. The participants exhibited limited knowledge about adverse outcomes of GDM for both mothers and neonates. Despite a high level of education among respondents, awareness levels remained low. Information on GDM was primarily derived from mass media and personal networks, while healthcare providers were reported as the least utilized source. Maternal and neonatal outcome awareness was notably insufficient, highlighting a need for comprehensive education and awareness programs.

We recommend the enhancement of healthcare provider involvement and educational initiatives to address the knowledge gap about GDM. Further research, including longitudinal studies to track knowledge levels about GDM over time, qualitative investigations into factors influencing awareness, and evaluation of targeted educational interventions, is also recommended. Conducting such research would deepen our understanding of GDM awareness and inform more effective educational strategies.
